# Controlled synthesis of Ni/CuO_x_/Ni nanowires by electrochemical deposition with self-compliance bipolar resistive switching

**DOI:** 10.1038/srep23069

**Published:** 2016-03-15

**Authors:** Kyuhyun Park, Jang-Sik Lee

**Affiliations:** 1 Department of Materials Science and Engineering, Pohang University of Science and Technology (POSTECH), Pohang 790–784, Korea

## Abstract

We demonstrate synthesis of Ni/CuO_x_/Ni nanowires (NWs) by electrochemical deposition on anodized aluminum oxide (AAO) membranes. AAO with pore diameter of ~70 nm and pore length of ~50 μm was used as the template for synthesis of NWs. After deposition of Au as the seed layer, NWs with a structure of Ni/CuO_x_/Ni were grown with a length of ~12 μm. The lengths of 1^st^ Ni, CuO_x_, and 2^nd^ Ni were ~4.5 μm, ~3 μm, and ~4.5 μm, respectively. The Ni/CuO_x_/Ni device exhibits bipolar resistive switching behavior with self-compliance characteristics. Due to the spatial restriction of the current path in NW the Ni/CuO_x_/Ni NW devices are thought to exhibit self-compliance behaviour. Ni/CuO_x_/Ni NWs showed bipolar resistive changes possibly due to conducting filaments that are induced by oxygen vacancies. The reliability of the devices was confirmed by data retention measurement. The NW-based resistive switching memory has applications in highly scalable memory devices and neuromorphic devices.

Resistive switching random access memory (RRAM) is based on electrical resistance changes by applied electrical biases. They are candidates for next-generation non-volatile memories because of their simple metal/insulator/metal structure, low power consumption, high stacking density, fast switching speed, high scalability, simple fabrication, and multistate behaviors^1^,^2^,^3^,^4^,^5^. Resistive switching behavior has been reported in transition metal oxides, such as TiO_2_^6^, ZnO^7^, HfO_2_^8^ and NiO^9^.

To improve the device performance and to increase the density of memory, the size of the devices must be reduced. Such reduction can be studied by using low-dimensional nanomaterials such as nanowires (NWs), nanotube, or nanodots. Low-dimensional materials have different characteristics from bulk and thin-film materials due to nanoscale sizes, since the materials are confined in low dimensions and have very high surface-to-volume ratios. These nanomaterials have unique chemical, physical, or optical behavior that makes them candidates for use in nanoscale device applications. The resistive switching characteristics of nanostructure materials, such as Cu_2_O^10^,^11^, NiO^12^, Co_3_O_4_^13^, Zn_2_SnO_4_^14^ and ZnO NWs^15^, was investigated.

Currently, bottom-up approaches have been used to synthesize various nanomaterials, e.g. nanodots, nanowires, and nanotubes^11^,^16^,^17^. Bottom-up methods have many advantages such as fast processing, simple fabrication steps, production of highly-uniform layers, and low cost. Bottom-up methods require a template on which to grow the nanomaterials. Electrochemical deposition (ECD) is one of the bottom-up processes and a low-cost process to fabricate nanomaterials on anodized aluminum oxide (AAO) membranes on large scale at room temperature (RT)^18^,^19^,^20^. AAO membranes are commonly used as such templates because the depth, shape, pore diameter, and periodic pattern of AAO can be controlled by changing the anodizing voltage and time^21^,^22^. CuO_x_ NWs have been shown to exhibit RRAM characteristics, but only resistive switching layer was synthesized using AAO template^11^. In this study, we fabricated complete metal/oxide/metal NWs and characterized the electrical properties. Due to the spatial restriction of the current paths in NWs the Ni/CuO_x_/Ni NW devices exhibit self-compliance bipolar resistive switching behavior.

## Results and Discussion

**Figure 1:**
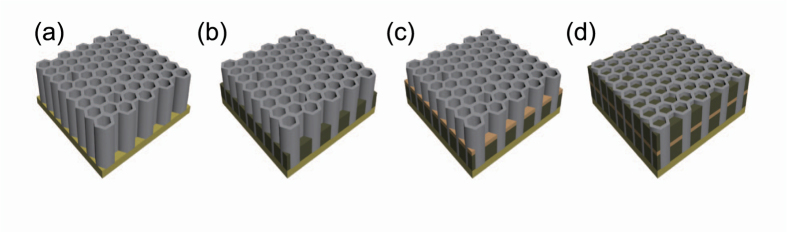
Schematic synthesis processes for Ni/CuO_x_/Ni NW array. (**a**) Deposition of Au seed layer on one-side of AAO template, (**b**) Ni growth by ECD, (**c**) CuO_x_ growth by ECD on bottom Ni, and (**d**) Ni growth by ECD on bottom CuO_x_/Ni.

The synthesis process of Ni/CuOx/Ni NWs is schematically illustrated in Fig. 1. The details about the synthesis methods of NWs can be found in Methods.

**Figure 2:**
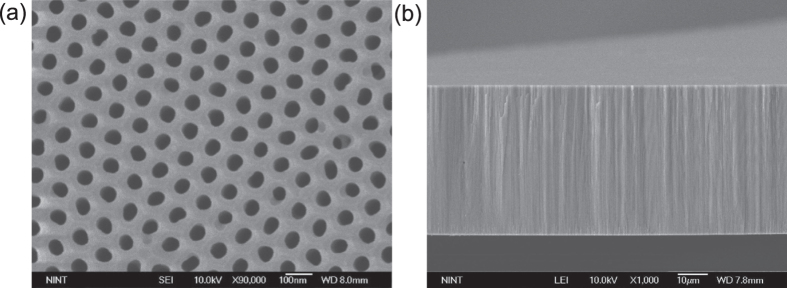
SEM images of AAO nanotemplates. (**a**) Plane-view and (**b**) cross-sectional view of AAO nanotemplates.

**Figure 3:**
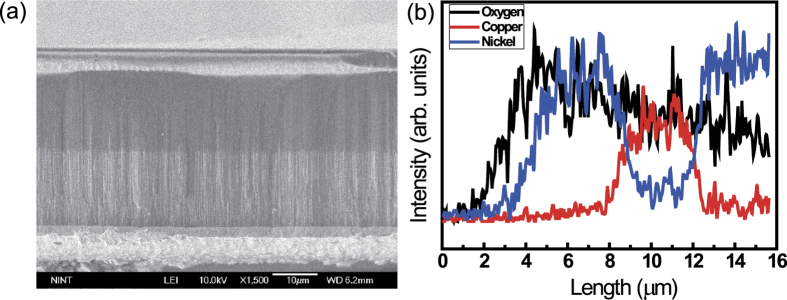
Synthesis of Ni/CuO_x_/Ni NWs. (**a**) Cross sectional SEM image of Ni/CuO_x_/Ni NW arrays in AAO nanotemplates and (**b**) EDS data of Ni/CuO_x_/Ni NW arrays in AAO nanotemplates.

The AAO template (Fig. 2(a)) had pore size ~70 nm and inter-pore distance ~110 nm, and was ~50 μm thick (Fig. 2(b)). We used it as the template on which to fabricate Ni/CuO_x_/Ni NW RRAM devices. After Ni, CuO_x_, and Ni NW growth, total NW length was ~12 μm (Fig. 3(a)). The lengths of the 1^st^ Ni, CuO_x_, and 2^nd^ Ni layers were ~4.5 μm, ~3 μm, and ~4.5 μm, respectively (Fig. 3(b)). Energy dispersive spectroscopy (EDS) data were obtained from a vertical cross section of AAO (Fig. 3(b)). EDS data confirmed the well formation of Ni/CuO_x_/Ni layer on AAO template. The NWs are synthesized using well-organized AAO templates, so the dimension of NWs, i.e. diameter and length, is found to be very uniform. The diameter of NWs is the same as the diameter of AAO pores since NWs are grown from bottom-up growth by ECD. There is some distribution of NW lengths, but this is because there are some broken NWs during removal of AAO templates and/or centrifugation of NWs for device fabrication.

In this experiment, Ni and CuO_x_ were deposited using ECD process. The Ni existed in the solution in the form of monovalent, positive ions (Ni^+^). When current is applied, the Ni^+^ ions react with electrons (e^−^) and are changed to metallic Ni at the cathode side. The CuO_x_ consisted of CuO and CuO_2_ formed simultaneously. The coexistence of Cu_2_O and CuO can be explained by the charge transfer reactions and diffusion. The reaction of copper ions with OH^−^ can yield Cu_2_O or CuO during ECD process. Among various materials that can be synthesized by ECD we chose Ni as the electrodes and CuO_x_ as the resistive switching materials. Both Ni and CuO_x_ can be easily deposited by ECD processes. In addition, the process is simple, inexpensive; can be done at low temperatures.

**Figure 4:**
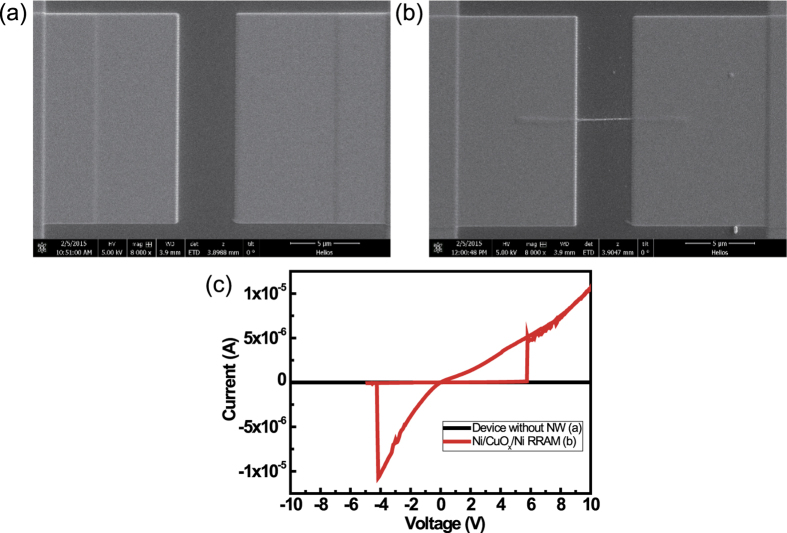
Fabricated device structures. (**a**) SEM image of Pt electrodes on SiO_2_ layer without NWs, (**b**) SEM image of Pt electrodes with NW devices, and (**c**) *I-V* characteristics of samples (**a**) and (**b**).

Electrical properties of the Ni/CuO_x_/Ni NWs were measured with Pt electrodes in voltage sweep mode (DC). The voltage was applied at one Pt electrode and the other Pt electrode was grounded. To compare Pt/Ni/CuO_x_/Ni/Pt with Pt/SiO_2_/Pt, Pt electrodes without Ni/CuO_x_/Ni NWs were fabricated on the SiO_2_ substrate (Fig. 4(a)). The gap between Pt electrodes was 4 μm in both Pt/Ni/CuO_x_/Ni/Pt and Pt/SiO_2_/Pt. The current−voltage (*I*–*V*) curves of Pt/Ni/CuO_x_/Ni/Pt (Fig. 4(b)) and Pt/SiO_2_/Pt were measured at RT in air. The *I*–*V* curves without NWs under voltage sweep mode from −10 V to 10 V demonstrate that the Pt/SiO_2_/Pt does not show RRAM characteristic in this voltage range, and that it is an insulator (Fig. 4(c), black line). However Pt/Ni/CuO_x_/Ni/Pt devices showed the RRAM characteristic, i.e., the Ni/CuO_x_/Ni device exhibited the resistive switching behavior (Fig. 4(c), red line).

**Figure 5:**
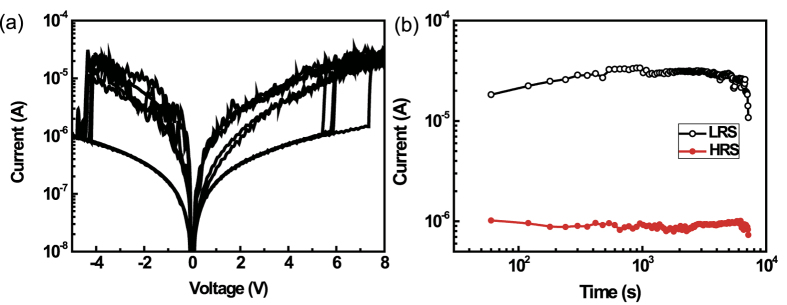
Electrical properties of Ni/CuO_x_/Ni NW devices. (**a**) Typical resistive switching characteristics of Ni/CuO_x_/Ni NW device. (**b**) Data retention characteristics of Ni/CuO_x_/Ni NW device.

*I*-*V* characteristics of a Ni/CuO_x_/Ni device were measured using the Pt deposited at the end of the NW (Fig. 5a). When a DC voltage was swept from 0 to 10.0 V with compliance current (CC) of 1 mA, a sudden increase of current was observed at 5.2 ~ 7.2 V; this is the set process. Afterwards, when the direct voltage bias was swept from 0 to −5 V, the resistance of the NW returned to the high resistance state (OFF-state); this the reset process and the corresponding voltage is the reset voltage *V*_RESET_ (in this device −4.5 ≤ *V*_RESET _≤ −4 V). When the set and reset voltages are of opposite polarity, the RRAM device is called a bipolar switching device. The operation voltage is found to be high in this device structure. This is due to the length of CuO_x_. The device is based on NWs and the length of CuO_x_ is about 3 μm. It is believed that the operation voltage can be reduced if the NW length for resistive switching layer is reduced and/or the properties of CuO_x_ layer is engineered by controlling the process parameters, for example, the current density, pH, deposition temperature, etc. Ni/CuO_x_/Ni device shows self-compliance behavior in the set part, which means that the devices do not need CC. Data retention by the Ni/CuO_x_/Ni NW device was measured at RT for 2 h. It maintained high resistance state (HRS) or low resistance state (LRS) for 2 h (Fig. 5b).

Resistive switching in the NWs is thought to be associated with migration of oxygen vacancies (V_o_s)^23^,^24^,^25^ and Joule heating under the electrical field^11^. In the initial state of Ni/CuO_x_/Ni NW device before set or reset process the CuO_x_ layer contains many V_o_s. When positive bias is applied, V_o_s diffuse from anode to cathode (grounded electrode) and form metallic filaments under the electric field during the set process^26^,^27^. At the same time, electrons move from cathode to anode. The metallic Cu nanoparticles included in CuO_x_ layer can increase the diffusion mobility of V_o_s and increase the local electric field. Furthermore, the one-dimensional nature of NWs in this confined configuration provides a linear path for V_o_s diffusion, and can give a stable switching characteristic to the device. During the reset process heat released by Joule heating disrupts the conducting paths.

The range of V_SET_ (5.2 to 7.2 V) was wider than the range of V_RESET_ (−4.5 to −4.0 V). The difference is related to the nature of the processes. The set process is affected by the competition among different filamentary paths at randomly uncertain filament sizes, orientations, and locations, so the process has a large random component^11^. In contrast, during the reset process, existing filaments rupture when Joule heating exceeds the energy required for filament rupture, so rupture happens in approximate unison. Therefore a narrow V_RESET_ distribution is expected.

The device was fabricated with a metal/oxide/metal structure in a single NW using ECD process. In the Ni/CuO_x_/Ni NW RRAM, both the metal (Ni) and oxide (CuO_x_) layers were ~70 nm in diameter. This small diameter constrains the filament path along which the current flows. Therefore the device shows the self-compliance current behavior. The CC is essential to keep the RRAM from failing permanently. However these devices show self-compliance current and do not need CC during the operation process. The reason to show self-compliance behavior is related to defect concentration/species in resistive switching layer. Further study is under way to investigate the exact nature to show the self-compliance behavior in our device structure.

We used ECD process, which is a ‘bottom-up’ technique, to fabricate the NW structure. Bottom-up processing can be used to synthesize smaller geometries. ECD process does not require the vacuum equipment and etching step; process time is short and the process step is simple in comparison to top-down techniques. This experiment shows the feasibility of NW RRAM devices fabricated using ECD process. In this experiment, metal/insulator/metal structured NWs could be scaled down to nanoscale dimension and exhibit RRAM characteristic with self-compliance behavior. The properties of oxide layers can be controlled by adjusting the current density, pH of solution, and/or deposition temperature during ECD process; NW diameter can be controlled by changing the dimension of AAO template. Furthermore NW lengths can be changed by controlling the deposition time. These properties suggest the possibility of controlling NWs for possible application to next-generation RRAMs.

## Conclusion

Ni/CuO_x_/Ni NWs that show RRAM characteristics were synthesized by ECD process in an AAO template. We demonstrated the feasibility of using ECD to produce Ni/CuO_x_/Ni NW RRAM devices. The devices showed bipolar RRAM characteristic and self-compliance behavior. Due to the spatial restriction of the current path in NW the Ni/CuO_x_/Ni NW devices exhibit self-compliance behaviour. Because of the self-compliance behavior, this device did not need CC during the measurement process. The resistive switching mechanism is related to migration of V_o_s, which form conductive filaments when an electric field is applied, whereas Joule heating may break the filaments. The range of set voltage *V*_set_ (5.2 ~ 7.2 V), which is wider than the range of reset voltage *V*_RESET_ (−4.5 ~ −4.0 V) because the set process, the formation of conducting filaments has a stochastic component. Ni/CuO_x_/Ni NW devices retained data for more than 2 h. In this study the feasibility Ni/CuO_x_/Ni NW in RRAM was demonstrated. Since it is possible to control the NW lengths, diameters, and CuO_x_ characteristics by controlling the deposition time, AAO diameters, and ECD process conditions, the technology presented in this work can be a good candidate for NW-based device applications.

## Methods

Ni/CuO_x_/Ni NWs were fabricated using ECD on AAO nanotemplate with a pore diameter of ~70 nm obtained by two-step anodization^28^. An Al (99.997%) sheet (2 × 10 cm^2^) was electro-polished twice in a perchloric acid HClO_4_/C_2_H_5_OH (1:4 in volume ratio) solution for 300 s at 7 °C. As the first anodization step, the Al sheet was anodized at 40 V in 0.3 M oxalic acid (H_2_C_2_O_4_) solution for 24 h at 7 °C. Then the AAO sheet was etched in a mixed solution of 0.2 M H_2_CrO_4_ and 0.4 M H_3_PO_4_ for 4 h at 60 °C. Then the second anodization was performed for 12 h. After the second anodization, the pores were widened in 0.1 M H_3_PO_4_ for 30 min at 30 °C. The Al sheet was sliced into 1 × 2 cm^2^ samples, which were immersed in supersaturated HgCl_2_ solution. To obtain an AAO layer, the barrier layer was removed by immersion in 0.1 M H_3_PO_4_ for 40 min at 30 °C. This process yielded AAO with a thickness of 50 μm and a pore diameter of ~70 nm.

Ni/CuO_x_/Ni NWs were formed on the AAO template (Fig. 1). A 120-nm Au layer was deposited by E-beam evaporation in a vacuum of ~5 × 10^−6 ^Torr to form a seed layer. Ni was deposited by ECD on the seed layer of the AAO under constant current of 4 mA for 2 h at 20 °C in aqueous electrolyte containing 8.4 × 10^−2 ^M nickel chloride hexahydrate, 1.6 M nickel sulfamate tetrahydrate, and 0.3 M boric acid^29^,^30^. The ECD system used in this study has a conventional three-electrode cell that uses a graphite counter electrode. Magnetic stirring was applied to improve the diffusion of metal ions at the cathode. The samples were rinsed with DI water, then CuO_x_ NWs were synthesized from an aqueous solution prepared from 0.6 M copper (II) sulfate pentahydrate (Aldrich) and 3 M lactic acid (Aldrich). After adding a certain amount of 5 M NaOH, the solution was left overnight with magnetic stirring. The stable solution was then adjusted to desired pH 9^31^,^32^,^33^. CuO_x_ was deposited by ECD on the Ni layer of the AAO under constant current of 4 mA for 30 min at 60 °C. The sample was rinsed with DI water, then Ni was electrodeposited on the CuO_x_ layer in the AAO under the constant current of 4 mA for 2 h at 20 °C. Finally, the AAO layer was dissolved by immersing the samples in 1 M NaOH aqueous solution for 4 h at RT; Ni/CuO_x_/Ni NWs were remained thereafter. The samples were rinsed in DI water, then centrifuged for five times to spread the NWs.

To measure electrical resistive switching properties Ni/CuO_x_/Ni NWs were drop-cast on a 100-nm-thick SiO_2_/Si substrate. For the tip contact in the probe station, first Pt electrodes (15 × 15 μm^2^) were deposited at the end of the NW and again larger Pt (30 × 30 μm^2^) pad was deposited at end of the electrode. Focused ion beam was used for Pt electrode deposition.

## Additional Information

**How to cite this article**: Park, K. and Lee, J.-S. Controlled synthesis of Ni/CuO_x_/Ni nanowires by electrochemical deposition with self-compliance bipolar resistive switching. *Sci. Rep.* **6**, 23069; doi: 10.1038/srep23069 (2016).
